# Effects of Narrowband Ultraviolet B Therapy on the Skin Barrier in a Murine Model of Dry Skin

**DOI:** 10.1111/phpp.70016

**Published:** 2025-04-11

**Authors:** Sumika Toyama, Yayoi Kamata, Atsuko Kamo, Mitsutoshi Tominaga, Kenji Takamori

**Affiliations:** ^1^ Juntendo Itch Research Center (JIRC), Institute for Environmental and Gender‐Specific Medicine Juntendo University Graduate School of Medicine Urayasu Chiba Japan; ^2^ Faculty of Health Care and Nursing Juntendo University Urayasu Chiba Japan; ^3^ Department of Dermatology Juntendo University Urayasu Hospital Urayasu Chiba Japan

1

Ultraviolet (UV)‐based therapies, such as psoralen plus UVA and narrowband UVB (NB‐UVB), are effective for certain dermatological disorders [1, 2]. We reported NB‐UVB suppressed itch in a dry skin mouse model by normalizing the expression balance of axon guidance molecules in epidermal keratinocytes [2]. In this study, we examined the effects of NB‐UVB in a dry skin mouse model produced by acetone‐ether treatment.

The protocol for cutaneous barrier disruption was performed as described, with modifications [3]. As the timeline in Figure 1A shows, hair on the rostral back of institute of cancer (ICR) mice (purchased from SLC, Shizuoka, Japan) was shaved off 2 days before AE (acetone and diethyl ether) treatment commenced on Day 1. AE treatment involved a cotton swab (2 × 2 cm) soaked in a 1:1 mixture of acetone and diethyl ether applied to the shaved area for 15 s twice daily for five consecutive days plus the morning following the fifth day, Day 6. Immediately following the final AE treatment, the shaved skin was irradiated with 10 mJ/cm^2^ NB‐UVB. Additionally, starting from prior to the first AE treatment on Day 1 and daily through Day 10, transepidermal water loss (TEWL) and stratum corneum (SC) hydration in the treated area were measured using Tewameter TM210 and Corneometer CM825 (Courage & Khazawa, Cologne, Germany), respectively (Figure 1A). Skin samples were harvested on Day 10 after the final TEWL and SC measurements, 4 days following NB‐UVB irradiation, and frozen sections were prepared and sliced at a thickness of 5 μm. Slides were air‐dried for at least 1 h, then blocked with PBS containing 2% bovine serum albumin (Sigma‐Aldrich, St. Louis, MO, USA), 0.2% Triton X‐100, and 5% normal donkey serum (Merck Millipore Corp., Darmstadt, Germany) at room temperature for 1 h. Sections were incubated overnight at 4°C with primary antibodies (Table 1), washed with PBS containing 0.05% Tween 20, and incubated with an Alexa Fluor 488‐conjugated secondary antibody (1:300 dilution; Thermo Fisher Scientific, Waltham, MA, USA) at room temperature for 1 h. Samples were counterstained with 4′,6‐diamidino‐2‐phenylindole (Wako Pure Chemical Industries). Fluorescence intensity was quantified using a BZ‐X800 microscope and analyzer (Keyence Corp., Osaka, Japan) to assess barrier‐related protein and tight junction molecule expression.

**FIGURE 1:**
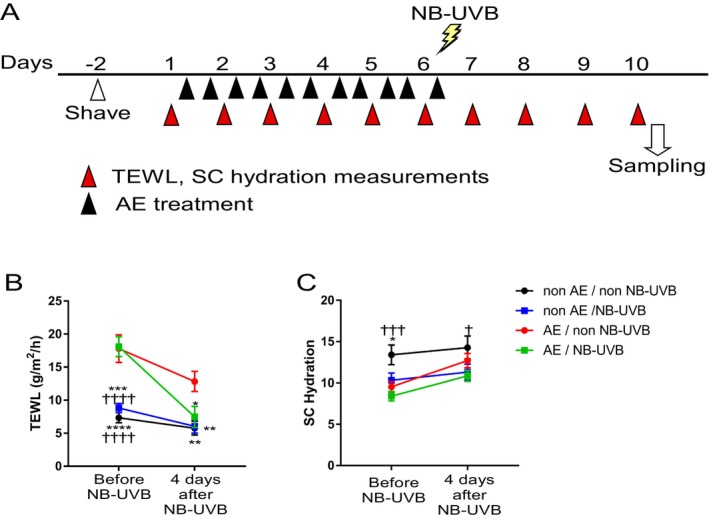
Effects of NB‐UVB on dry skin. (A) Experimental scheme. (B) Effects of treatment on transepidermal water loss (TEWL). (C) Effects of treatment on stratum corneum (SC) hydration. *N* = 11–15. *p < 0.05, **p < 0.01, ***p < 0.001, and ****p < 0.0001 indicate significant differences from AE‐treated mice, ^†^p < 0.01, ^†††^p < 0.001 and ^††††^ *p* < 0.0001 indicates a significant difference from AE + NB‐UVB‐treated mice in a two‐way ANOVA with Tukey's multiple comparison test. Data are presented as the mean ± SEM of three independent experiments.

**TABLE 1:** Primary antibody information.

Target	Company	Cat no.	Dilution
Filaggrin	Biolegend	905804	1/100
Loricrin	Biolegend	905104	1/100
Claudin‐1	Invitrogen	PA5‐16833	1/200
Involucrin	Biolegend	PRB‐140C	1/500
Occludin	Cell Signaling	91131	1/100
ZO‐1	ProteinTech	21773‐1‐AP	1/1000

AE treatment increased TEWL over time, which was significantly reduced by NB‐UVB irradiation (Figure 1B). SC hydration significantly decreased with AE treatment but slightly improved after NB‐UVB irradiation (Figure 1C). Among barrier‐related proteins and tight junction molecules, filaggrin (Figure 2A) and loricrin (Figure 2B) expression was significantly higher in the AE + NB‐UVB group than in the AE‐only group. Claudin 1 and involucrin expression were unaffected by AE treatment or NB‐UVB irradiation (Figure 2C,D). Occludin expression slightly increased following AE treatment but decreased after NB‐UVB irradiation (Figure 2E). ZO‐1 expression showed a slight increase after NB‐UVB irradiation (Figure 2F).

**FIGURE 2:**
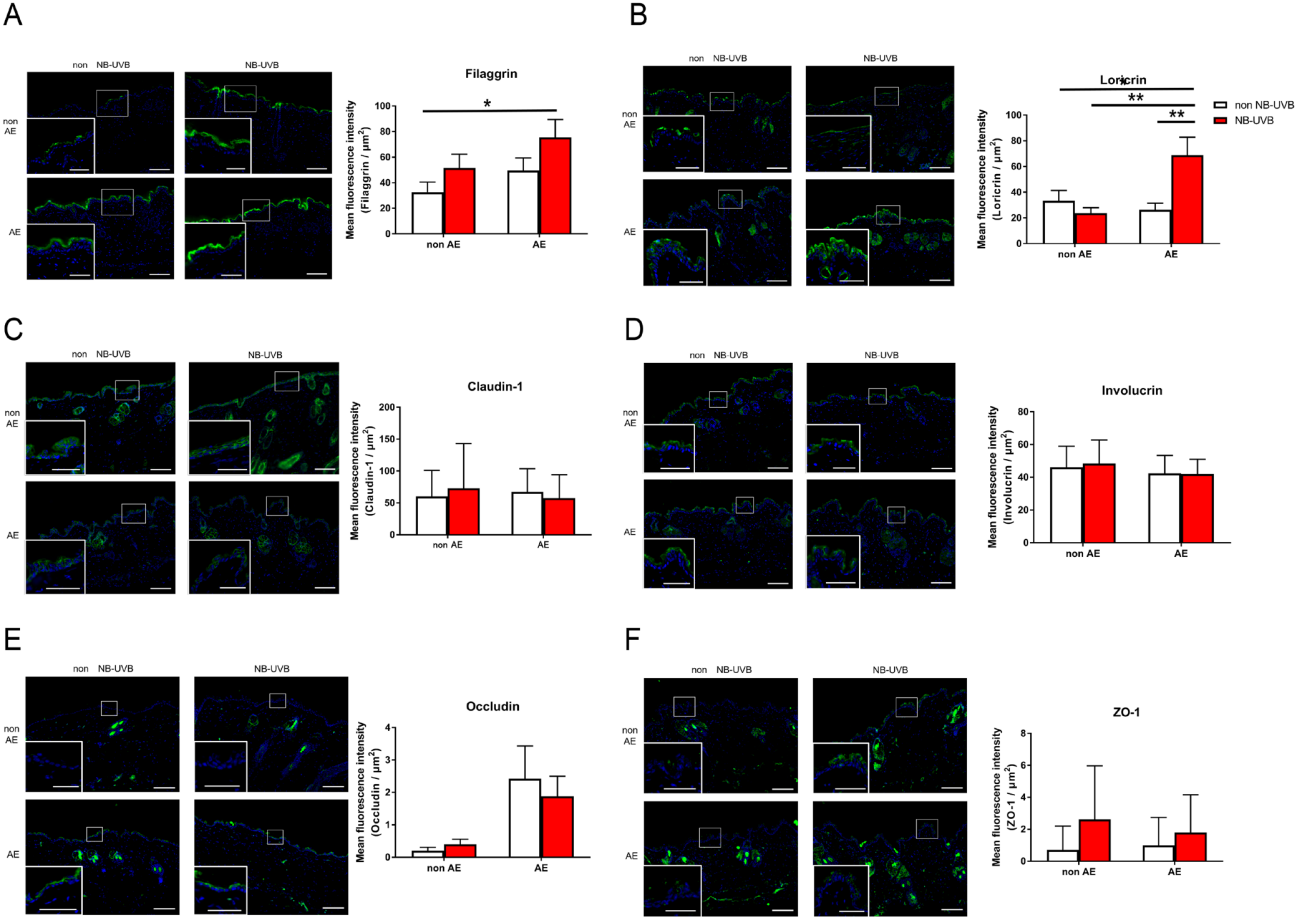
Effects of NB‐UVB on tight junctions and barrier molecules in a dry skin model. (A) Immunostaining of filaggrin in naïve mice (upper left panel), NB‐UVB‐treated mice (upper right panel), AE‐treated mice (lower left panel), and AE + NB‐UVB‐treated mice (lower right panel) (left). Semi‐quantitative fluorescence intensity of filaggrin (right). (B) Immunostaining of loricrin in naïve mice (upper left panel), NB‐UVB‐treated mice (upper right panel), AE‐treated mice (lower left panel), and AE + NB‐UVB‐treated mice (lower right panel) (left). Semi‐quantitative fluorescence intensity of loricrin (right). (C) Immunostaining of claudin‐1 in naïve mice (upper left panel), NB‐UVB‐treated mice (upper right panel), AE‐treated mice (lower left panel), and AE + NB‐UVB‐treated mice (lower right panel) (left). Semi‐quantitative fluorescence intensity of claudin‐1 (right). (D) Immunostaining of involucrin in naïve mice (upper left panel), NB‐UVB‐treated mice (upper right panel), AE‐treated mice (lower left panel), and AE + NB‐UVB‐treated mice (lower right panel) (left). Semi‐quantitative fluorescence intensity of involucrin (right). (E) Immunostaining of occludin in naïve mice (upper left panel), NB‐UVB‐treated mice (upper right panel), AE‐treated mice (lower left panel), and AE + NB‐UVB‐treated mice (lower right panel) (left). Semi‐quantitative fluorescence intensity of occludin (right). (F) Immunostaining of ZO‐1 in naïve mice (upper left panel), NB‐UVB‐treated mice (upper right panel), AE‐treated mice (lower left panel), and AE + NB‐UVB‐treated mice (lower right panel) (left). Scale bars = 100 μm. Enlarged view scale bars = 50 μm. Semi‐quantitative fluorescence intensity of ZO‐1 (right). *p < 0.05 and **p < 0.01 indicate significant differences in a two‐way ANOVA with Tukey's multiple comparison test. Data are presented as the mean ± SEM of three independent experiments.

A single exposure to NB‐UVB partially restored skin barrier function in AE‐treated dry skin by inducing filaggrin and loricrin expression. Previous studies have shown NB‐UVB irradiation increases filaggrin expression [4]. UVB exposure activated the ROS‐MAPK/AP‐1/TGF‐β‐Smad signaling cascade [5], in which AP‐1, a transcription factor, regulates filaggrin and loricrin expression [5, 6]. These findings suggest NB‐UVB irradiation enhanced the epidermal ROS‐MAPK/AP‐1/TGF‐β‐Smad signaling cascade, leading to increased filaggrin and loricrin expression and barrier restoration. Filaggrin, a key SC structural protein and natural moisturizing factor precursor, contributes to skin barrier function [7]. Thus, NB‐UVB‐induced filaggrin expression may mitigate TEWL by enhancing the moisturizing effects of AE treatment on dry skin. Loricrin is essential for keratinocyte maturation, but not SC permeability. Therefore, NB‐UVB‐induced loricrin expression may improve skin barrier flexibility and partially normalize its function.

Since loricrin protects against UVB‐induced damage, its expression may have been upregulated in response to NB‐UVB irradiation [8]. In psoriasis patients, NB‐UVB has been shown to increase loricrin gene expression and restore normal keratinization [9]. In this study, samples were collected 4 days after NB‐UVB irradiation, when its effects on loricrin expression were likely at their peak. However, NB‐UVB irradiation did not increase loricrin expression in the non‐AE‐treated group (Figure 2B). This discrepancy suggests a negative feedback mechanism under normal loricrin expression levels prevents NB‐UVB‐induced upregulation. NB‐UVB is highly safe and effective, with no serious side effects reported even with long‐term use [10]. Although this study examined a single exposure, long‐term NB‐UVB may further enhance skin barrier function. However, potential long‐term risks, such as photocarcinogenesis and photoaging, should be considered when planning treatment [10].

In conclusion, our findings indicate NB‐UVB irradiation of dry skin increases filaggrin and loricrin expression, at least partly restoring their protective effects and improving skin barrier function.

## Conflicts of Interest

The authors declare no conflicts of interest.

## Data Availability

The data that support the findings of this study are available from the corresponding author upon reasonable request.
